# A Suppressive Antagonism Evidences Progesterone and Estrogen Receptor Pathway Interaction with Concomitant Regulation of *Hand2*, *Bmp2* and ERK during Early Decidualization

**DOI:** 10.1371/journal.pone.0124756

**Published:** 2015-04-21

**Authors:** Ana C. Mestre-Citrinovitz, Veronika Kleff, Griselda Vallejo, Elke Winterhager, Patricia Saragüeta

**Affiliations:** 1 Instituto de Biología y Medicina Experimental, IByME-Conicet, Buenos Aires, Argentina; 2 Institut für Anatomie, Universaetsklinikum Duisburg-Essen, Essen, Germany; 3 Institut für Molekulare Biologie, Universaetsklinikum Duisburg-Essen, Essen, Germany; Michigan State University, UNITED STATES

## Abstract

Progesterone receptor and estrogen receptor participate in growth and differentiation of the different rat decidual regions. Steroid hormone receptor antagonists were used to study steroid regulation of decidualization. Here we describe a suppressive interaction between progesterone receptor (onapristone) and estrogen receptor (ICI182780) antagonists and their relation to a rescue phenomenon with concomitant regulation of *Hand2*, *Bmp2* and p-ERK1/2 during the early decidualization steps. Phenotypes of decidua development produced by antagonist treatments were characterized by morphology, proliferation, differentiation, angiogenesis and expression of signaling molecules. We found that suppression of progesterone receptor activity by onapristone treatment resulted in resorption of the implantation sites with concomitant decrease in progesterone and estrogen receptors, PCNA, KI67 antigen, DESMIN, CCND3, CX43, *Prl8a2*, and signaling players such as transcription factor *Hand2*, *Bmp2* mRNAs and p-ERK1/2. Moreover, FGF-2 and *Vegfa* increased as a consequence of onapristone treatment. Implantation sites from antagonist of estrogen receptor treated rats developed all decidual regions, but showed an anomalous blood vessel formation at the mesometrial part of the decidua. The deleterious effect of onapristone was partially counteracted by the impairment of estrogen receptor activity with rescue of expression levels of hormone steroid receptors, proliferation and differentiation markers, and the induction of a probably compensatory increase in signaling molecules *Hand2*, *Bmp2* and ERK1/2 activation compared to oil treated controls. This novel drug interaction during decidualization could be applied to pathological endometrial cell proliferation processes to improve therapies using steroid hormone receptor targets.

## Introduction

The uterus provides a unique and dynamic physiological model in which cellular proliferation, differentiation and apoptosis occur in a spatiotemporal and cell-specific manner during pregnancy. Decidualization comprises a rapid remodeling of the uterine stromal compartment resulting in a morphological and functional transformation [1, 2]. This complex shift in the cell program builds the decidua, a specialized compact tissue responsible for successful implantation. The decidua has a critical role to ensure proper maternal-fetal interactions and guides trophoblast invasion, placental orientation and development [3]. The transdifferentiation process of stromal cells is coordinated by the priming effect of the steroid hormones, Estradiol (E) and Progesterone (P); and the signaling interaction with the implanting blastocyst [2, 4]. Although numerous molecules of the signaling pathway necessary for decidual development have been identified, the hierarchical instructions that coordinate ovarian hormone actions with the embryo-uterine dialogue are not well understood.

The decidua presents different morphological and functional areas: the antimesometrial decidua (AM) is characterized by compacted and round cells and is the site where the embryo implants; the mesometrial decidua (M), a less compact area, is important for the development of the vasculature and the ingrowth of the placenta. These two differently differentiated zones, AM and M, are clearly separated by the junctional zone (J), which maintains a stromal character. Similarly, the decidual area underneath myometrium (UM) remains also undifferentiated conserving endometrial glands [5].

We previously described the role of progesterone receptors (PR), estrogen receptors (ER), in ERK activation during *in vivo* decidualization [6]. We studied the changes in PR, ER and activated ERK (p-ERK) localization during the late kinetic of pregnancy and after treatment of pregnant rats with pure progesterone or estrogen antagonist alone and in combination (subcutaneous-sc- injection) and with the ERK1/2 phosphorilation inhibitor PD98059 (intraperitoneal-ip- injection). We showed that PR and ER participate in growth and differentiation of the different rat decidual regions and suggested a new function of p-ERK1/2 in regulating expression levels of ER α, thereby keeping the proliferation capacity of stromal cells and limiting the differentiation process in specified regions of decidual tissues. In this paper we describe the relation between PR, ER α, their signaling pathways and a novel drug interaction during the initial steps of decidualization by steroid hormone antagonists administration at 5 and 6 dpc of rat pregnancy. Phenotypes of decidua development produced by antagonist treatments were characterized by morphology, proliferation, differentiation, angiogenesis and expression of signaling molecules.

## Materials and Methods

### Reagents

Hormone antagonists: Antiprogestin Onapristone (ONA) (ZK 98299, Bayer Schering, Germany); Antiestrogen faslodex (ICI182780, ICI) (Tocris Bioscience, Bristol, UK). Solutions: RIPA buffer (50 mM Tris/HCl, 150 mM NaCl, 1% NP-40, 0.25% Na-deoxycholate, 1 mM EDTA, 0.1% SDS) supplemented with EDTA-free Complete Protease Inhibitor Cocktail and PhosSTOP Phosphatase Inhibitor Cocktail (Roche, Mannheim, Germany); Phosphate-buffered saline (PBS; 1.3 mM NaH_2_PO_4_H_2_O, 9.7 mM Na_2_HPO_4_, 145.4 mM NaCl; pH 7.4); Citrate Buffer (8.2 mM Sodium Citrate, 1.8 mM Citric Acid; pH 6.0). Bradford protein assay kit (Bio-Rad Laboratories, California, USA). RNeasy Midi-Kit (Qiagen, Hilden, Germany). Power SYBR Master Mix (Applied Biosystems), Hematoxylin (BIOPUR diagnostics, Buenos Aires, Argentina), Eosin (Cicarelli Laboratorios, Buenos Aires, Argentina). Streptavidin peroxidase complex (Millipore, Billerica, NA, USA), 3.3 diaminobenzidine (DAB) (Dako, Glostrup, Denmark). Bovine serum albumin (BSA) (Sigma-Aldrich, St. Louis, MO, USA). The following primary antibodies were used: rabbit polyclonal C20 anti-hPR (1:1000); rabbit polyclonal H190 anti-hPR (1:100); rabbit polyclonal MC20 anti-mER α (1:2000, 1:100), rabbit polyclonal C14 anti-rERK2 (1:1000); mouse monoclonal PC10 anti-rPCNA (1:1000), rabbit polyclonal C16 anti-hCyclinD3 (1:500) and rabbit polyclonal anti-FGF-2 (1:200) were bought from Santa Cruz Biotechnology Inc. Rabbit polyclonal anti-CX43 (1:3000) was bought from Sigma-Aldrich; rabbit monoclonal 14C10 anti-hGAPDH (1:3000) and rabbit monoclonal D13.14.4E against hERK1/2 phosphorylated at Thr202/Tyr204 (1:2000, 1:200) were obtained from Cell Signaling Technology; mouse monoclonal M0724 anti-DESMIN (1:1000) was purchased from Dako and rat polyclonal anti-CD31 (1:20) from Dianova. Rabbit polyclonal ab15580 anti-KI67 (1:200) was bought from Abcam. Biotinilated secondary antibodies E0432 anti-rabbit (1:200), E0433 anti-mouse (1:100) and anti-rat (1:200) immunoglobulins were obtained from Dako Inc. Horseradish peroxidase (HRP) linked secondary antibodies NA934 anti-rabbit (1:3000) and NA931 anti-mouse (1:3000) immunoglobulins were purchased from GE Healthcare Life Sciences.

### Animal care

All animal experiments were approved by the Institutional Animal Care Committee of the Instituto de Biología y Medicina Experimental and the Universitaetsklinikum Duisburg-Essen and were carried out in accordance with local and state regulations for research with animals.

Adult female Wistar rats (Charles River Laboratories, Germany) were housed under controlled temperature (22°C ± 1°C, atmospheric humidity of 55% ± 10%) and lighting (12 hs light: 12 hs dark cycles) conditions. The rats were fed with standard pellet food and ad libidum water.

### Pregnancy

Mating was performed overnight and the following morning sperm was evaluated by vaginal smear. The day of sperm finding was designated as day 0 post coitum (dpc). The 4 dpc pregnancies were confirmed by flushing the uterus and analyzing blastocyst presence. The 5, 6, 7, 8, 9, 10 and 20 dpc pregnancies were confirmed by the presence of implantation sites (ISs).

### PR and ER antagonist treatments

The antiprogestin Onapristone (ONA) was dissolved in benzilbenzoat (20 mg/ml). For subcutaneous injection, 0.5 or 1 mg ONA were adjust to a final volume of 150 or 300 μl with peanut oil, respectively. The antiestrogen ICI was dissolved in ethanol (20 mg/ml), the volume corresponding to 0.5 mg was adjust to 500 μl with peanut Oil and injected subcutaneously. Rats (n = 3) were injected with 1 or 0.5 mg ONA (ONA), 0.5 mg ICI (ICI), or 1 mg ONA + 0.5 mg ICI (ONA+ICI) per day at days 5 and 6 pc. Control animals (n = 3) were injected with vehicle (oil). Implantation sites were collected at day 7, 10 and 20 pc. The label ¨ONA¨ in the figures represents 1 mg ONA treatment, unless specified.

### Tissue Collection

Rats were killed by cervical dislocation under isofluran-anesthesia. Non-pregnant (NP), 2 and 4 dpc pregnant uterine horns were removed and cut into pieces and frozen in liquid nitrogen for subsequent western blot analysis. Implantation sites from 6, 7, 8, 9, 10 and 20 dpc uterine horns were dissected. ISs were collected and frozen in liquid nitrogen for western blot analysis and total RNA extraction, or fixed in 4% paraformaldehyde (PFA) for morphological and immunohistochemical analysis. For 20 dpc morphological analyses, placentas (P) and embryos (E) were collected and weighted. The relation P weight to E weight (P/E) was calculated.

### Western Blot Analysis

Protein extracts were obtained from tissue samples by homogenization (Polytron PT-MR 300, Brinkmann Instruments, NY, USA) in RIPA buffer supplemented with EDTA-free Complete Protease Inhibitor and Phosphatase Inhibitor Cocktail. Protein concentration was determined by Bradford protein assay kit. SDS-PAGE and immunoblot analyses were performed to detect DESMIN, CYCLIN D3 (CCND3), PCNA, FGF-2, CX43, Progesterone receptor (PR), Estrogen receptor alfa (ER α), GAPDH, phosphorylated ERK1/2 proteins (p-ERK1/2) and ERK2. Detection was achieved with chemiluminescent substrate using X-ray films (Kodak, Stuttgart, Germany). The band intensities were measured with ImageQuant 3.3 program (Amersham Pharmacia Biotech, Arlington Heights, IL). GAPDH and ERK2 were use as loading controls. Relative values of protein levels were express as fold change of treatment intensity arbitrary units over its respective control (NP and oil).

### RNA extraction and qRT-PCR

Total RNA was isolated from 7 dpc ISs (n3) using the RNeasy Midi-Kit according to the manufacturer’s instructions. The RNA was reverse transcribed and *β-Actin*, *Prl8a2*, *Vegfa*, *Hand2* and *Bmp2* levels were quantified by quantitative Real Time-PCR (qRT-PCR) using the system of detection ABI PRISM 7500 (Applied BiosytemsQ7). Cycle threshold (CT) values were calculated with 7500 System Software Sequence Detection System 1.3 (Applied BiosytemsQ7) and absolute quantifications were carried out using known quantities of standard copy DNA (cDNA), resulting values were expressed in femtograms (fg). *β-Actin* was used as an internal control. Relative values of mRNA levels were express as fold change of treatment fg over its respective control (oil). Amplification of cDNA was performed at 60°C using primers listed in S1 Table.

### Histochemistry

After fixation in 4% PFA, ISs were dehydrated in a graded series of alcohol, and- embedded in paraffin. Serial sections of 7 μm were deparaffinized, rehydrated in a graded series of alcohol and stained with hematoxylin-eosin (H&E). Photographs were taken with an Axiophot microscope (Zeiss, Jena, Germany). The area of different regions from the ISs was delimited as shown in S1 and S2 Figs and quantified with ImageJ 1.43 software (National Institute of Health, Bethesda, Maryland, USA). The extent of the different decidual regions was calculated in relation to the area of the whole decidua, designated as 100%.

### Immunohistochemistry

For immunostaining, 7 μm paraffin sections were deparaffinized and rehydrated in a graded series of alcohols. After rinsing with PBS, the endogenous peroxidase was blocked and the sections were incubated with 0.5% bovine serum albumin for 30 min. The sections were incubated overnight at 4°C with the specific antibodies, rinsed in PBS (3 x 5 min), followed by incubation with a biotinylated secondary antibody for 30 min at RT and for further 30 min with a streptavidin-peroxidase complex. Staining was visualized with 3.3 diaminobenzidine (DAB). For the detection of CD31, KI67 and PR, the slices were boiled for 20 min in Citrate Buffer and left at room temperature (RT) for 40 min before blocking with BSA. KI67 staining was counterstained with hematoxylin. Controls were performed omitting the primary antibody.

### KI67 quantification

Pictures from the different decidual areas were taken from KI67 immunohistochemistry (IHC) staining. KI67 positive signal was visualized as brown nuclei while non-proliferative cells were seen as blue (hematoxylin staining). The percentages of proliferative cells per area of the decidua were quantified blinded to the treatment condition with the plugin Cell counter from ImageJ 1.43 software. The calculated percentage represents the number of KI67 positive cells over total number of cells per field (100 percent). A minimum of 3 fields was quantified for each area analyzed in each IS.

### Quantification of blood vessels and lacunas

Blood lacunas and CD31 positive areas were quantified with ImageJ 1.43 software. The CD31-IHC images taken with the Axiophot microscope were adjusted to grey scale and then a threshold was applied. The pixels with intensity above the threshold were turned into black while the pixels with intensity below the threshold were turned into white. In the final image CD31 positive staining and blood lacunas were black over a white background. The blood vessels and lacunas area was calculated in relation to the area of the whole image, designated as 100 percent. Only vessels and lacunas from the J and M area (J+M) were quantified.

### Statistical Analysis

Analysis of variance was used for statistical testing, followed by Tukey Multiple Comparison Test. For frequency analysis in tables contingency analysis followed by chi-square test was performed. Student's t-test was used in S2 Fig Differences were considered significant if P < 0.05. Statistical Analysis was carried out with GraphPad Prim 4.0 (GraphPad Software Inc., La Jolla, CA, USA).

## Results

### Changes in the morphology and gene expression of markers of the decidua after ER and PR antagonists effects

**Fig 1:**
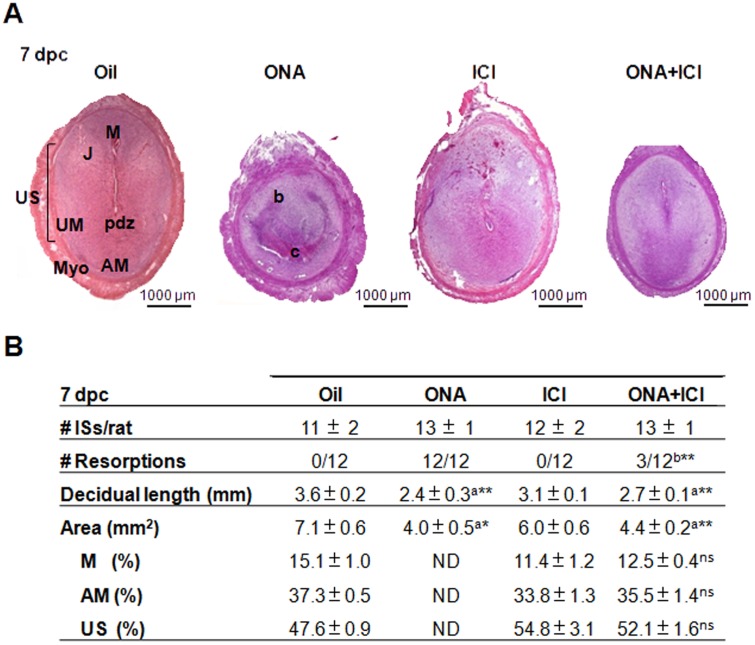
Morphology effects of ONA, ICI and ONA+ICI on 7 dpc implantation sites after 5 and 6 dpc consecutive injections. A) Figures show H&E staining of a representative 7 dpc IS from Oil, ONA, ICI and ONA+ICI treated rats. B) Quantitative analysis. Table shows the number of implantation sites/rat, the number of resorptions/total number of ISs analyzed, the mean ± SEM length and total area of decidua and the percentage of tissue areas corresponding to the different regions within the ISs relative to the area of total decidua. Images analyzed are shown in S1 Fig Data represent mean fold change ± SEM from at least 3 independent rats/treatment. *, P < 0.05; **, P < 0.01; ***, P < 0.001; a, statistical differences v. Oil; b, statistical differences v. ONA; ns, no statistical differences v. Oil; ND, No development of decidua;. AM, antimesometrial decidua; M, mesometrial decidua; J, junctional zone; UM, under myometrium; US, undifferentiated stroma; pdz, primary decidual zone; Myo, myometrium; b, border of resorpted IS; c, centre of resorpted IS. mm, millimeters; mm^2^, square millimeters. Bar = 1000 μm.

**Fig 2:**
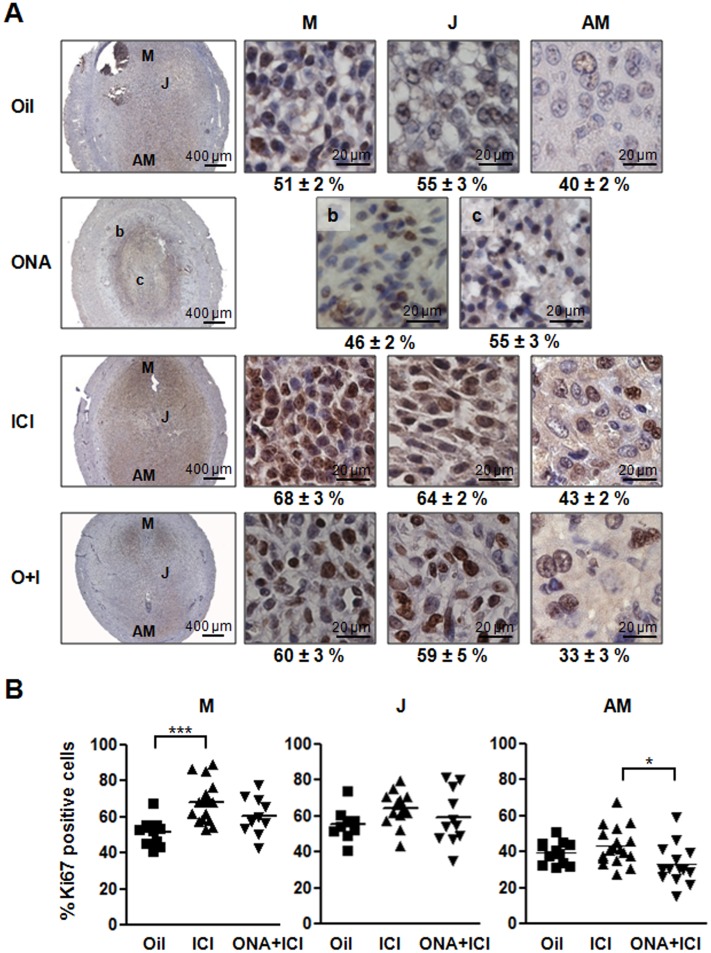
Effects of antagonist treatments on proliferation. Samples from 7 dpc Oil, ONA, ICI or ONA+ICI treated rats were analyzed for KI67 protein expression. A) Details of KI67 immunostaining of M, J and AM zones from a representative 7 dpc IS from Oil, ICI and ONA+ICI treated rats; and the border (b) and central (c) zones from a representative ONA-treated IS. Mean percentages ± SEM of positive KI67 cells are shown under each picture. B) KI67 quantification. Each point in the graph represents the percentage of the proliferative cells per field. Three fields per IS area were count and a minimum of 3 ISs were analyzed for each treatment. Fig 2A data represent mean fold change ± SEM from at least three independent rats/treatment, a minimum of 1 IS/rat was analyzed. *, P < 0.05; **, P < 0.001. O+I, ONA+ICI; M, mesometrial decidua; J, junctional zone; AM, antimesometrial decidua; b, border area of resorpted IS; c, center area of resorpted IS. Magnification bars = 400 μm; magnification bar = 20 μm.

Our previous study using the antagonists of PR (Onapristone, ONA) and ER (ICI) during early pregnancy between days 6 and 8 pc [6] indicated that the action of both ovarian hormones would be more effective and pronounced on the decidualization process if applicated earlier in pregnancy. It should also restrict time and manner of approaching molecular signaling pathways. Thus we treated pregnant rats with progesterone receptor antagonist or estrogen receptor antagonist alone and in combination one day earlier than started above [6] on days 5 and 6 pc and collected the implantation sites (ISs) on day 7 pc. In contrast to treatment on days 6 and 7 pc ONA 1 mg/rat/day given one day earlier caused resorption of all ISs. ONA treatment produced a regression in the tissue with a shrinkage of all decidual tissues, avoiding the differences between AM, M and J observed in oil treated ISs (Figs 1A and 2). The ONA treated ISs showed residues of decidual tissue without an embryo (Fig 1 and S1 Fig). As 1 mg/rat/day caused total resorption of embryos, we reduced the ONA dose to 0.5 mg/day/rat with the same application protocol. The lower dose did not interfere with the implantation and decidualization process and resulted in normal ISs, with properly developed decidual areas (S2 Fig), indicating that lower PR activity might be sufficient for the decidualization but drastically lose effectiveness under a still undefined limit.

ICI-treated ISs developed all decidual regions, AM, M and J, with a more compacted development of M (Figs 1A and 2), but showed an enhanced effect on the blood vessel formation at the mesometrial part (M) of the decidua (Fig 1A and S1 Fig).

Surprisingly the combined action of ONA+ICI, which downregulated the action of both hormones, reverted the embryo resorptions induced by ONA alone (Fig 1A and S1 Fig) and produced the same cellular phenotype as oil treated animals (Fig 2). However, though all decidual compartments were appropriately developed, the ISs were obviously smaller (38 ± 7%) compared to control (Fig 1 and S1 Fig). When counting the resorption sites as successful implantations, the number of implantation sites/pregnant rat does not differ between the different treatments (Fig 1B). This result was expected, considering that the time schedule of treatments used did not interfere with the early implantation process [7].

Longitudinal decidual length, different decidual areas (AM: antimesometrial, M: mesometrial and US: undifferentiated stroma) (indicated in S1 and S2 Figs) of ISs were quantified for all treatments. The ratio between AM and M to the complete decidua did not change in ICI, 0.5 mg ONA or ONA+ICI (Fig 1B and S2 Fig).

To analyze in detail the decidual proliferation dependency on ER and PR action we studied two proliferation markers, KI67 antigen and PCNA (Fig 2 and S3 Fig). ONA-resorpted ISs showed a lower percentage of KI67 positive nuclei cells (Fig 2A). Two areas were distinguished in the resorpted ISs: a disorganized central area (c) and a border zone (b). The former (c) had more KI67 positive nuclei than the ones in the latter (b). Regarding the proliferation per functional decidual area, only ICI treatment increased proliferation in the mesometrium (M), while ONA+ICI decreased the proliferation in the antimesometrial area (AM) in respect to ICI treatment (Fig 2B). It is worth noting that the distribution of the proliferation percentages of Oil treated rats showed lower dispersion than the percentages of ICI and ONA+ICI treated rats (Fig 2B). On the other hand, protein levels of PCNA were significantly lower in ISs treated with ONA compared with controls (S3 Fig). ICI and ONA+ICI showed only a tendency to diminish the expression of PCNA proliferation marker (S3 Fig). These results are in accordance with a significantly reduced decidualization observed in ONA and ONA+ICI treatment, and with a slightly reduced developed decidual area in ICI treated rats (Fig 1B). While ER antagonism seems to interfere only slightly with proliferation activity, impairment of PR action was most drastic and resulted in complete resorptions, confirming that progesterone is the stimulating hormone for endometrial stromal proliferation.

**Fig 3:**
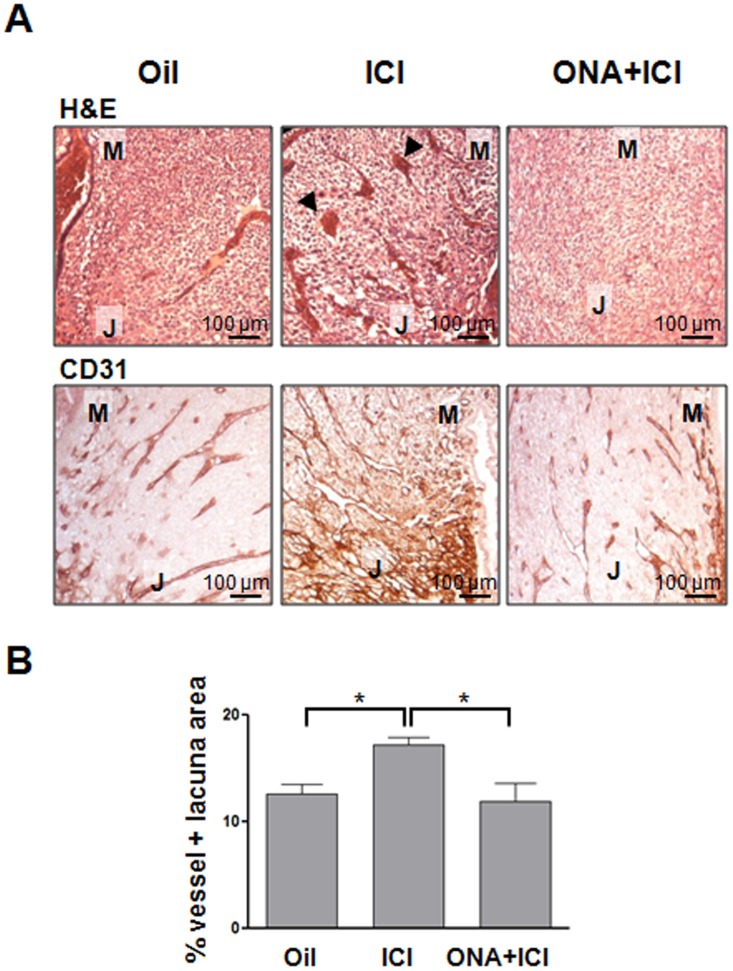
Effects of antagonist treatments on decidual vessels formation. A) Detail of junctional zone (J) of H&E (upper panel) and CD31 immunostaining (lower panel) of a representative 7 dpc ISs from Oil, ICI and ONA+ICI. B) Quantification of vessel and lacunas structures of J+M areas. Data represent the mean ± SEM of the percentage of blood vessels and lacunas area from H&E images relative to total J+M area from at least three independent rats/treatment. *, P < 0.05. M, mesometrial decidua; J, junctional zone. Bar = 100 μm. Arrows heads indicate blood lacunas.

For a detailed view of the vasculature in the ISs of animals treated with the antagonists, we performed immunohistochemistry against CD31, a marker for endothelial cells [8] (Fig 3A). CD31 expression confirmed the disorganization in the vasculature of the J+M area with numerous dilated vessels and red cells in lacunas already observed in the histology of ICI treated ISs (Fig 1A, S1 Fig and Fig 3A). CD31 positive area and lacunas were quantified, showing an increase in ICI-treated ISs in relation to Oil and ONA+ICI treatments (Fig 3B). There were no significant differences between the vessels and lacunas area of Oil and ONA+ICI-treated rats (Fig 3B). These results support a clear ICI effect on vasculogenesis in the mesometrial and junctional area. CD31 IHC of ONA ISs showed plenty of newly-formed vessels in the border area of the resorption, indicating a possible vascular reorganization process (S4 Fig).

**Fig 4:**
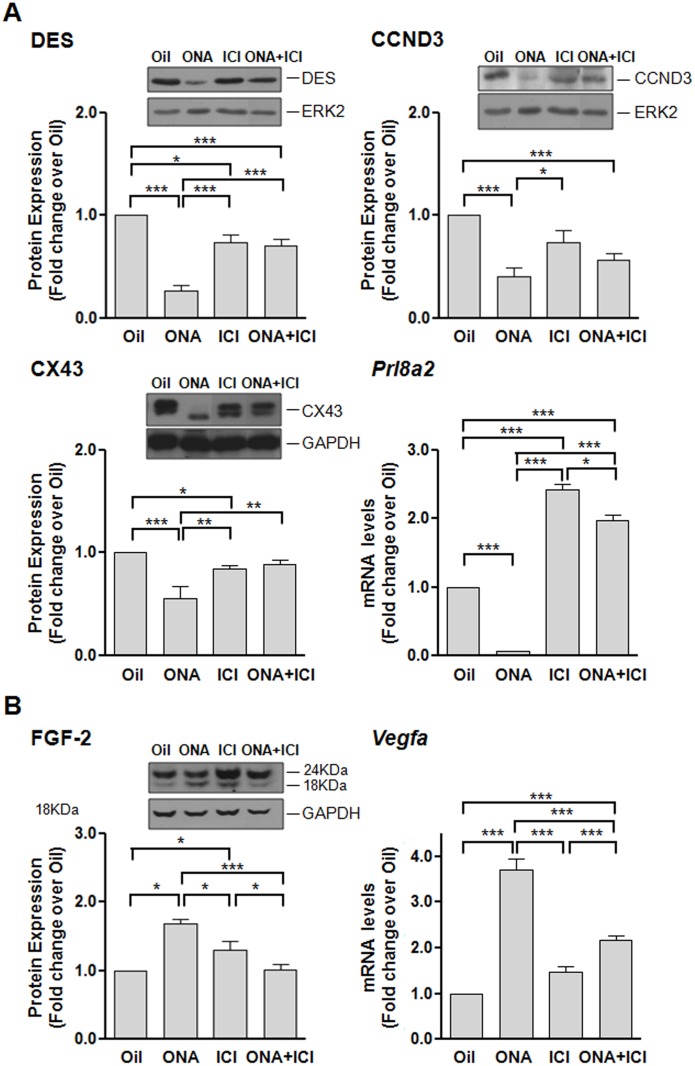
Effects of antagonist treatments on differentiation and vascularization. Samples from 7 dpc Oil, ONA, ICI or ONA+ICI treated rats were analyzed for protein and mRNA expression of differentiation and vascularization markers. A) Differentiation markers: protein levels of CCND3, DESMIN, CX43 and mRNA levels of *Prl8a2*. B) Vascularization markers: protein levels of FGF-2 and mRNA levels of *Vegfa*. Each treatment protein levels relative to GAPDH or ERK2, or mRNA levels relative to *β-Actin*, were divided by the corresponding Oil-treated value. Data represent mean fold change ± SEM from at least three independent rats/treatment. Insets show pictures of a representative western blot. *, P < 0.05; **, P < 0.01; ***, P < 0.001.

We further characterized the decidualization process by studying decidual differentiation (Fig 4A) and vascularization (Fig 4B) markers and their dependency on the ER and PR action. For changes in decidual differentiation after ER and PR antagonists treatment we investigated DESMIN (DES), a classical structural decidual marker [9], and CYCLIN D3 (CCND3), a polyploidization and terminal differentiation marker [10], Connexin 43 (CX43), a gap junction protein highly enriched in decidua and critical for decidual differentiation and angiogenesis [11–13], and mRNA expression of *Prl8a2*, the decidual/trophoblast prolactin-related protein abundantly expressed in decidua [14] (Fig 4A). During the progress of early pregnancy we observed that protein expression of Desmin and CCND3 markers increased in parallel with development of decidua, reaching a maximum at 6–7 dpc for DES and at 7 dpc for CCND3 expression (S5 Fig). In the case of antagonist treated animals, ONA alone produced a remarkably reduced Desmin and CCND3 expression, and a smaller reduction in the combined treatment (Fig 4A). CX43 was downregulated by ONA and ICI alone treatments (Fig 4A). *Prl8a2* was downregulated in ONA resorpted sites, and was upregulated by ICI and ONA+ICI treatments (Fig 4A). These results indicate that the smaller size of ONA+ICI ISs in relation to oil treatment is due not only to a reduce proliferation but also to a loss of differentiation capacity resulting in suboptimal developed ISs.

Regarding vascularization, we measured FGF-2 [15] protein and *Vegfa* [16] mRNA (Fig 4B). Both markers of angiogenesis were upregulated in ISs from ONA treated rats (Fig 4B). ICI treatment showed upregulation of FGF-2 while ONA+ICI treated rats showed upregulated *Vegfa* mRNA expression (Fig 4B). The higher levels of FGF-2 and *Vefga* observed in ONA treated animals could reflect the reorganization in morphology and vasculature of the implantation chambers of ONA treated animals induced by the resorption process. CD31 immunohistochemistry of ONA resorptions confirmed this vascular reorganization (S4 Fig). The higher levels of FGF-2 and *Vefga* observed in ICI treated animals could be due to a phenomenon exclusively restricted to vessel formation. The low dose of ONA did not produce changes in differentiation and vascularization marker expression (S2 Fig).

### Rescue effect of reduced PR action by additional ER antagonist

**Fig 5:**
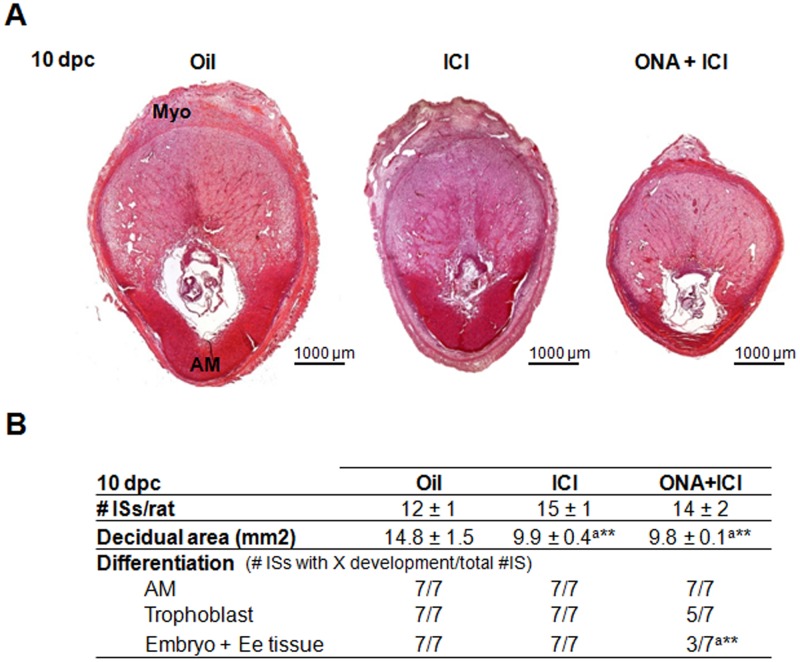
Morphology effects of ICI and ONA+ICI on 10 dpc rat implantation sites after 5 and 6 dpc consecutive injections. A) Pictures show H&E staining of a representative IS treated with Oil, ICI and ONA+ICI at 10 dpc. B) Quantitative analysis of 10 dpc ISs. Table shows the number of implantation sites/rat, the mean ± SEM of total area of decidua and the proportion of ISs with AM, trophoblast and embryo and extra-embryonic tissue (Embryo + Ee tissue). ONA+ICI ISs analyzed are shown in S6 Fig Data represent mean fold change ± SEM from at least three independent rats/treatment, a minimum of 3 IS/rat was analyzed. **, P < 0.01; a, statistical differences v. Oil; Differentiation (Number of ISs with development of the different areas over total number of IS analyzed). AM, antimesometrial decidua; Myo, myometrium; X represents: AM, Trophoblast or Embryo + Ee tissue; mm^2^, square millimeters. Bar = 1000 μm.

To further study the ICI rescue of ONA-induced ISs resorption, we analyzed different time points (10 dpc and 20 dpc) after administration of ICI alone and in combination with ONA, allowing pregnancy to continue nearly until labour. At 10 dpc we observed that ICI produced normal and smaller ISs than oil treated rats (Fig 5). ONA+ICI produced heterogeneous results summarized in Fig 5 and S6 Fig All the ISs analyzed (7/7IS/3rats) showed normal differentiated decidua as confirmed by antimesometrial cells morphology, 71.4 percent (5/7IS/3rats) revealed trophoblast differentiation and 42.9 percent (3/7IS/3rats) exhibited embryonic and extra-embryonic tissue development, including proper placenta development, not correctly orientated in some cases (Fig 5 and S6 Fig). Nevertheless, ICI and ONA+ICI-treated ISs were significantly smaller than ISs of 10 dpc oil treated rats (Fig 5B). At day 20 pc development of ICI and 0.5 mg ONA treated ISs continued to be normal, but the relationship between placenta and embryo sizes increased with an increment in placenta size and a decrease in embryo size (Table 1 and S2 Fig). All 20 dpc embryos in every ONA+ICI treated rat analyzed were resorpted (S7 Fig).

**Table 1:** Effects of ICI and ONA+ICI on 20 dpc implantation sites after 5 and 6 dpc consecutive injections.

20 dpc	Oil	ICI	ONA+ICI
**# ISs/rat**	13 ± 1	14 ± 1	11 ± 3^**R**^
**Placenta weight (g)**	0.64 ± 0.01	0.74 ± 0.02^a^ ***	ND
**Embryo weight (g)**	4.25 ± 0.06	3.96 ± 0.05^a^ ***	ND
**P/E relation**	0.15 ± 0.01	0.19 ± 0.01^a^ ***	ND

Quantitative analysis of 20 dpc embryos and placenta. Table shows the number of implantation sites/rat, the mean ± SEM of total area of decidua, the Placenta and Embryo weight and the relation of Placenta weight to Embryo weight. Data represent mean fold change ± SEM from at least three independent rats/treatment. g, grams. ND, No development of placenta and embryo.

*** P < 0.001.

^a^ statistical differences v. Oil.

^R^ only resorptions were found.

### Signaling pathways in ER and PR antagonist decidualization effects

**Fig 6:**
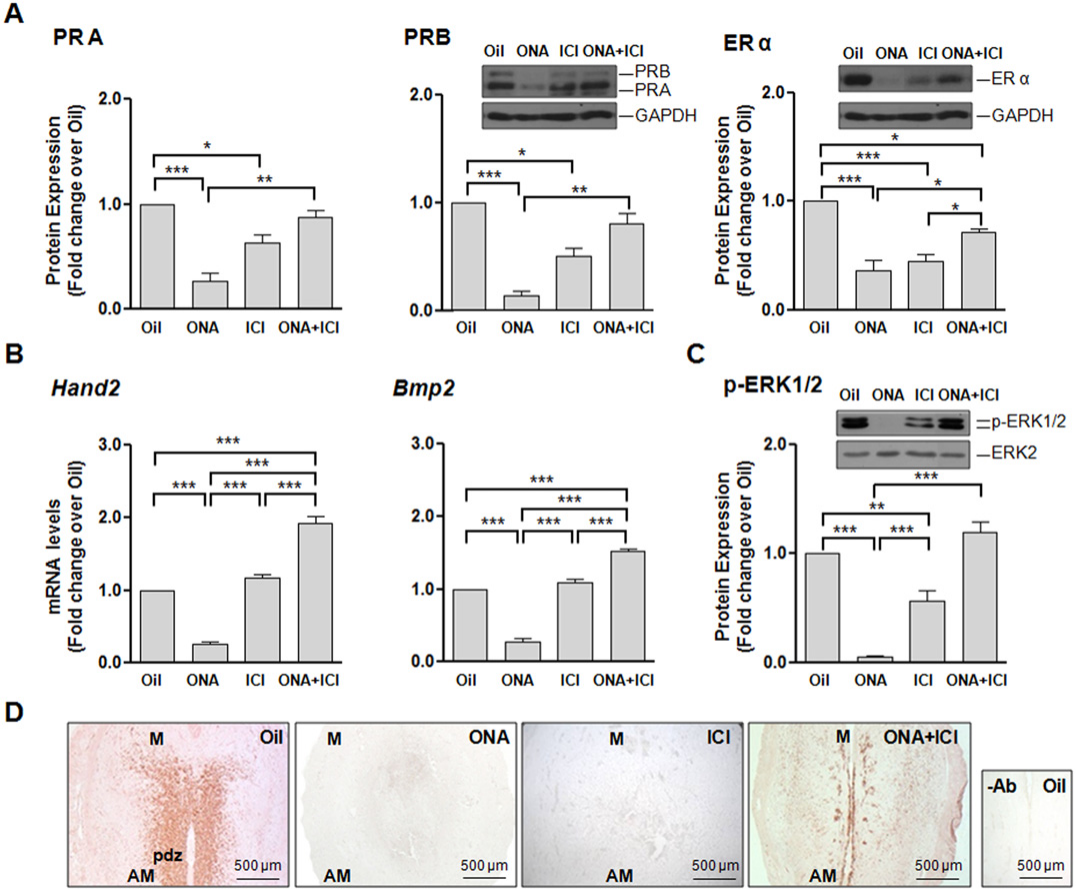
Effects of antagonist treatments on PR and ER expression and signaling pathways. Samples from 7 dpc Oil, ONA, ICI or ONA+ICI treated rats were analyzed for protein and mRNA expression of steroid receptors and different genes related to known decidualization signaling pathways. A) Protein expression levels of PRA, PRB and ER α were analyzed by western blot. B) *Bmp2* and *Hand2* mRNA expression levels were analyzed by qRT-PCR. C-D) Variations in the levels of ERK1/2 activation were analyzed by western blot and Immunohistochemistry. Pictures in D) show staining for p-ERK1/2 on paraffin sections of implantation sites from 7 dpc treated animals. For all figures, each treatment protein level value relative to ERK2 or mRNA level value relative to *β-Actin* was divided by the corresponding Oil-treated level. Data represent mean fold change ± SEM from at least three independent rats/treatment, a minimum of 2 IS/rat was analyzed. Insets show pictures of a representative western blot. *, P < 0.05; **, P < 0.01; ***, P < 0.001. AM, antimesometrial decidua; M, mesometrial decidua; pdz, primary decidual zone; -Ab, IHQ without specific antibody. Bar = 500 μm.

**Fig 7:**
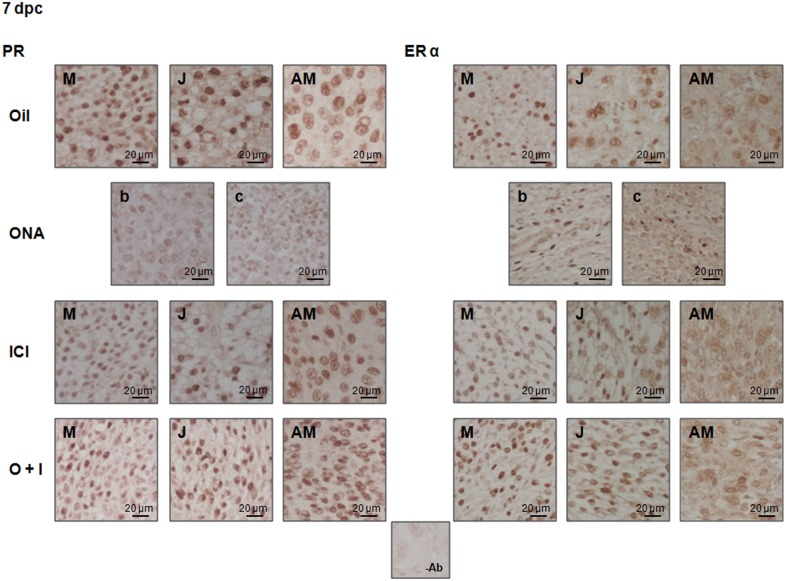
Effects of antagonist treatments on PR and ER α localization. IHC for PR and ER α from 7 dpc Oil, ONA, ICI and ONA+ICI treated ISs were analyzed for protein localization of steroid receptors. Pictures show a representative IS of each decidual area/treatment staining for PR and ERα on paraffin sections of implantation sites from 7 dpc treated animals. AM, antimesometrial decidua; M, mesometrial decidua; J, junctional zone; b, border area of resorpted IS; c, center area of resorpted IS;-Ab, IHQ without specific antibody. Bar = 20 μm.

In regard to the signaling pathways involved in the action of ovarian steroid receptors we analyzed the levels of PR and ER α during decidual differentiation. We found that ONA-induced resorptive ISs expressed low levels of both PR receptors but the combined action of ONA+ICI recovered the progesterone receptors to the levels of oil treated rats (Fig 6A). To note, the levels of ER α however were downregulated by the antagonist treatments alone and in combination when compared to controls but compared to single treatments ER α has recovered significantly (Fig 6A). The localization for PR and ER α were analyzed by IHC of both receptors in 7 dpc ISs treated with oil, ONA, ICI and ONA+ICI. All treatments presented positive signals for both receptors (Fig 7) as was previously described for 8 dpc [6]. PR was present in the cytoplasm and nuclei of J, M and AM cells (Fig 7). In AM PR positive signal appeared also in the periphery of the nuclei of AM differentiated cells (Fig 7). ER α localized in the cytoplasm and nuclei of AM, J and M cells. ICI alone and in combination with ONA treatments did not change steroid hormone receptors localization (Fig 7). ONA-induced resorpted ISs show low expression of PR and ER α in the b and c zones (Fig 7).

The mRNA expression of genes previously described as being involved on PR and ER signaling during decidualization—*Bmp2* [17], *Hand2* and FGF-2 [18]- was analyzed by qRT-PCR and western blot respectively. *Bmp2* showed a low expression in ONA 0.5 mg dose and this downregulation was enhanced in resorptions, ICI treated animals showed no changes in compared to controls, and the combined action of ONA+ICI upregulated *Bmp2* expression (Fig 6B and S2 Fig). Similarly, low levels of *Hand2* expression were observed in the resorption sites of ONA treated rats compared to normal 7 dpc ISs, nearly no change in ICI treated animals while the combined action of ONA+ICI revealed a significant increase (Fig 6B).

ONA 0.5 mg treated rats revealed no changes in PRA, PRB and ER α protein expression (S2 Fig). *Vegfa*, *Prl8a2* and *Hand2*, mRNAs were also not statistically different from oil treated rats (S2 Fig).

We analyzed the effect of the antagonist treatments on the levels of activated ERK1/2 (p-ERK1/2) by western blot and inmunohistochemistry (Fig 6C and 6D). The active isoform of ERK, as well as PRA and B, was downregulated by ONA and ICI treatments alone and rescued to control levels by ONA+ICI treatment (Fig 6C). ONA 0.5 mg treated rats revealed a diminution of p-ERK (S2 Fig). Immunodetection of activated ERK1/2 shows a strong signal in the primary decidual zone (pdz) and some nuclei staining in the junctional zone of oil-treated ISs at day 7 pc (Fig 6D). No signal for p-ERK1/2 in ICI treated ISs was detected and a re-established lower signal in ONA+ICI treated ISs (Fig 6D). These findings were confirmed by protein levels seen in western blot analysis (Fig 6C).

We needed to find out whether ICI treatment ERK1/2 inactivation leads to vasculature effect or whether ICI vasculature failure caused ERK1/2 inactivation.

To explore the relation between ERK1/2 activation and the degree of decidua development we analyzed p-ERK1/2 localization at 10 dpc. While in Oil treated rats the signal of p-ERK1/2 was restricted to the junctional area and to the area in contact with the developing placenta (S6 Fig), in ONA+ICI treated rats the signal spread toward the mesometrial side together with the presence of vessel formation, marked by CD31 expression (S6 Fig). This could imply that ERK1/2 activation during late decidua appears more meaningful for vasculature reorganization when both antagonists are present.

Taken together, *Bmp2*, *Hand2* and p-ERK1/2 significantly increased in ONA+ICI treated animals and thus could contribute to the rescue effect in decidual morphology and function, leading to the correct establishment of pregnancy.

## Discussion

The highlight of the present study is that loss of PR activity during early pregnancy, which resulted in the resorption of implantation sites, was partially rescued by the abolishment of ER action. The rescue, however, supported pregnancy only up to day 10 pc but further progress was impaired by a failure in decidua, embryo and placenta development. ONA could produce some toxic effects on implanted embryos, leading to embryo resorption during early pregnancy. When the ONA dose was diminished and the pregnancy continued to 20 dpc the effect was a significant reduction in embryo size and a significant increment in placenta size. Similar results were obtained in the case of 20 dpc ICI treatment. Maternal and embryo toxic effects could lead to a miscommunication affecting proper pregnancy develop [1, 3, 4].

The ratio of progesterone to estrogen receptors seems to be most important for appropriate decidualization. Even if both hormone receptors are blocked by their respective antagonists, the remaining activity seems adequate for reduced but proper decidualization. Apart from resorptions, the development of the regionalized decidua into different compartments is not impaired, a fact that points to a mutual influence of the ingrowing embryo and placenta on the structural organization of this tissue.

### PR- and ER-antagonist effects on PR and ER expression

**Fig 8:**
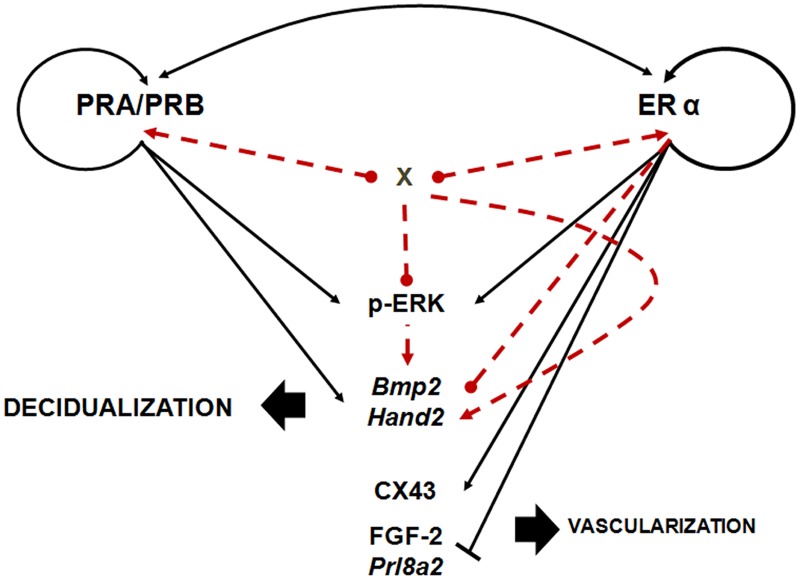
Scheme depicting interaction between PR and ER pathways in decidualization. Individual antagonist treatments evidenced PR and ER gene regulation pathways, meanwhile combined antagonist treatment highlights the existence of P and E receptors pathways interaction. ONA +ICI treatment restores ISs development by the activation of ¨compensatory pathways¨. This failsafe road could include regulation of *Hand2*, *Bmp2* expression, ERK activation and/or unidentified signaling molecules (X). Arrows heads, positive regulation; blunt heads, negative regulation; point head; undefined regulation; black fill line, interactions evidenced in this work; red dot line, pathways proposed.

Treatment with only one antagonist, whether ER or PR, resulted in a significant downregulation of PRA/B and ER α receptor expression, confirming the regulatory influence of each hormone on both receptors (Fig 8). Interestingly, simultaneous PR and ER antagonists administration, recover the hormone receptors status nearly to the level of the control. This phenomenon could contribute to the rescue of decidualization for it to proceed. These findings support the idea that not only the absence of PR results in a progesterone-dependent deregulation programming but also influences the regulation of ER action, which ends up in an abnormal development of the decidua leading to loss of the pregnancy.

### Downstream decidualization gene markers

The impaired decidual differentiation process is reflected by the changes in the expression of downstream decidualization-related genes such as *Hand2* [19], *Bmp2* [17], CCND3 [20], DESMIN, CX43 and *Prl8a2* [14].

DESMIN and CCND3 increase upon decidualization progression and reach their higher expression at 6–7 dpc respectively. Both markers are significantly reduced upon all treatment protocols and reflect the less differentiated decidual tissue observed in morphology. CX43 was also diminished by the treatment with both antagonists alone. ONA treatment presents a single and smaller band positive for CX43. This result could correspond to the non-phosphorylated (P0) form in ONA regressed decidua and the larger bands present and predominately in other treatment could correspond to P1 and P2 phosphorylated CX43 forms. ONA effect over CX43 isoforms in resorted ISs could be similar than the effects described by retinoic acid in human endometrial stromal cells [21]. Our results agree with CX43 expression stimulated by estrogen in preimplantation hormonal-mediated pathway versus embryo implantation-initiated pathway [11]. On the other hand high expression of *Prl8a2* in response to low activity of ER and PR reinforces its importance in pregnancy adaptation to physiological stressors [22].

Regarding proliferation, percent of KI67 nuclear antigen positive signal at 7 dpc decidual regions of Oil-treated ISs were in general lower than percents resulted in all other treatments. This result contrasted with what was expected considering that Oil-treated ISs were bigger than was observed for all other treatments, exception made of ICI that showed a similar size. These results could indicate that the stromal cell proliferation in Oil-treated rats took place before day 7 pc, while antagonist treatments (ICI and ICI+ONA) delayed the time of cell proliferation. This is not the case with ONA treatment, which produces a diminution of size by regression of the tissue. Levels of PCNA, another marker of cell proliferation, were significantly reduced only in the decidua of ONA treated rats. The equally slightly lower levels in the ICI or ONA+ICI treatments were not correlated to the differences in the size of decidual areas measured in H&E cross sections. PCNA levels could also reflect the higher endoreplicative state associated to a more differentiated state of Oil-treated ISs than that were observed in antagonist-treated ISs.

*Hand2 a*nd *Bmp2* followed a similar expression pattern after antagonist treatments with downregulation after antiprogestin, but an enhanced expression after antiestrogen and, specially, combined antagonist treatments. HAND2 protein has been shown to be localized in the uterine stroma, and increases during decidualization *in vitro* in mouse and human [19]. Suppression of *Hand2* by mRNA interference resulted in a reduced differentiation of decidua [19].

Recently, Bagchi et al. [23] have identified *Hand2* as a PR-regulated gene using the PR antagonist RU486 and by P-treatment of ovariectomized mice. Further investigations using a conditional knockout mouse revealed that mice lacking the progesterone-induced expression of *Hand2* had a continued stimulation of the estrogen pathway via the induction of ER α phosphorylation [18]. The dominance of ER α- signaling pathways is indicated by induction FGFs and the preservation of epithelial proliferation, which resulted in impaired implantation.

Moreover, HAND2 has been shown to mediate steroid effects for pregnancy progression [19]. Like in other studies, we found a downregulation of *Hand2* and an upregulation of FGF-2 in ONA-induced resorptive ISs and an upregulation under progesterone dominance in ICI treated animals. Inactivation of both, PR and ER action, further upregulates *Hand2*, which indicates a feedback mechanism to compensate for the loss of ovarian hormone receptors (Fig 8).

BMP2, which belongs to the multifunctional transforming growth factor superfamily, and its receptor has been shown to be stage specifically expressed in the decidua [24]. The use of the antiprogestin RU486 demonstrated that BMP2 belongs to the downstream target genes of P and is functionally involved in decidualization in mice and human [25]. Generating conditional deletion of *Bmp2*, Lee et al. [17] demonstrated that the observed infertility of mice is due to missing decidual response after blastocyst attachment. Furthermore, WNT-BMP2 pathway regulated by COUP-TFII has been previously described in the regulation of ER activity by P, showing its importance for blastocyst implantation and early pregnancy development [26, 27].

### PR and ER antagonist effects on angiogenesis

The absence of ER activity produces abnormalities in the sinusoidal vessel formation of the mesometrial decidua. Except for this abnormal vascularization, estrogen deprivation alone has only minimal effects on the decidual morphology and pregnancy progression. These results agree with previous studies that P is the ovarian hormone that plays a critical role during decidualization [28]. Furthermore, beyond embryo attachment, Das et al. [29] described a central role of locally synthesized estrogen during decidual angiogenesis. Thus, estrogen dominance in the case of progesterone antagonist treatment seems to support synergistically the resorption process by increasing the angiogenic potential. The abnormal vascularization observed in absence of ER could be partially explained by the high levels of FGF-2 resulting of PR-dominance after ICI treatment. Although FGF-2 expression has been previously associated to ER stimulation [30], other molecules were described as FGF-2 regulators in rat decidua-i.e. PRL [31]-. PRL8A2 secreted in the antimesometrial zone of decidua [14] could have a paracrine role regulating FGF-2 expression at the mesometrial side [31]. As shown in the present study *Prl8a2* mRNA is highly upregulated upon ICI treatment as well as in ONA+ICI treated rats. Together with the high levels of *Vegfa* found in these decidual tissues, both growth factors initiate a higher vascularization. Downregulation of CX43 in the presence of high *Vegfa* and FGF-2 could also contribute to disorganized vascularization. Whether HAND2 contributes to PRL expression regulation is still not clear [19]. In our previous study we found evidence that activation of ERK1/2 play a role in keeping the regionalized distribution of the decidualization process and thereby govern the distribution of the vessels [6]. However, as shown here, activated ERK1/2 does not seem to be a direct mediator of FGF-2 activity in our experimental conditions. On the other hand, the lack in ERK1/2 activation caused by ICI could contribute to disorganize the neo-angiogenesis. In addition, the upregulation of FGF-2 in the absence of p-ERK1/2 may be responsible for the observed enhanced and abnormal decidual angiogenesis (Fig 8).

In summary (Fig 8), the partial rescue produced by ER antagonist when injected together with PR antagonist reflects a suppressive interaction between both drugs. This hypothesis is supported by the suppressive effect of each hormone receptor on the opposite receptor and a tightly counteracting expression on the downstream target genes. On the bases of these findings, we propose that decidualization has evolved to an ‘optimal’ condition–i.e. maximizing growth decidual cell rate in a given condition and time. Reduced production of differentiation players such as *Bmp2* and *Hand2* in ONA treated animals results in an ineffective use of cellular resources and prevents pregnancy development (resorption). The loss of ERK activation needed for the correct regionalization of the IS contributes to the final deleterious result. The suboptimal scenario in response to ONA+ICI treatment could lead to the activation of compensatory ¨rescue¨ pathways (Fig 8). However, overproduction of the rescue molecules, *Hand2* and *Bmp2*, could lead to an excessive use of resources for decidualization at the expense of other cellular processes, i.e. proliferation, with a sub-optimal size of the implantation site in the case of ONA+ICI treatment. The observation that, under growth inhibition produced by ONA in presence of ICI, reduction in differentiation allows faster than ONA-alone-treatment growth of decidua suggests that decidual player levels are not optimally but functionally regulated under these hormonal conditions.

## Supporting Information

S1 Fig Morphology effects of ONA, ICI and ONA+ICI on 7 dpc implantation sites after 5 and 6 dpc consecutive injections. Pictures show H&E staining of 7 dpc IS from Oil, ONA, ICI and ONA+ICI treated rats analyzed and quantified in Fig 1B. Black lines define the decidual areas quantified. AM, antimesometrial decidua; M, mesometrial decidua; J, junctional zone; UM, under myometrium; US, undifferentiated stroma; Myo, myometrium; b, border area of resorpted IS; c, center area of resorpted IS. Bar = 500 μm.
(TIF)

S2 Fig Morphology effects and PR, ER and p-ERK levels of 0.5 mg ONA on 7 and 20 dpc implantation sites after 5 and 6 dpc consecutive injections. A) Pictures show H&E staining of a representative 7 dpc IS from 0.5 mg ONA treated rats. B) Quantitative analysis of ISs shown in A. Table shows the number of implantation sites/rat, the number of resorptions/total number of ISs analyzed, the mean ± SEM of length and mean ± SEM of total area of decidua and the percentage of tissue areas corresponding to the different regions within the ISs relative to the area of total decidua. Black lines define the decidual areas quantified. At least 3 independent ISs were quantified. C) Quantitative analysis of the effects of 0.5 mg ONA on 20 dpc implantation sites after 5 and 6 dpc consecutive injections. Table shows the number of implantation sites/rat, the mean ± SEM of total area of decidua, the Placenta and Embryo weight and the relation of Placenta weight to Embryo weight. D) Protein expression levels of PRA, PRB, ER α, CX43, FGF-2, DES, CCND3, GAPDH, ERK2 and activated ERK1/2 were analyzed by western blot and mRNA expression levels of *Vegfa*, Prl8a2, Bmp2 and *Hand2* analyzed by qRT-PCR. Data represent mean fold change ± SEM from at least three independent rats/treatment, a minimum of 2 IS/rat was analyzed. *, P < 0.05; ***, P < 0.001; a, statistical differences v. Oil; g, grams. AM, antimesometrium; M, mesometrium; J, junctional zone; UM, under myometrium; US, undifferentiated stroma; Myo, myometrium. Bar = 500 μm. mm, millimeters; mm^2^, square millimeters; g, grams.
(TIF)

S3 Fig Effects of antagonist treatments on proliferation. Samples from 7 dpc Oil, ONA, ICI or ONA+ICI treated rats were analyzed for protein expression of PCNA. In each treatment, protein levels of PCNA relative to ERK2 were divided by Oil-treated value. Data represent mean fold change ± SEM from at least three rats/treatment, a minimum of 2 IS/rat was analyzed. Insets show pictures of a representative western blot. *, P < 0.05.
(TIF)

S4 Fig Effects of ONA treatment on vessels formation. CD31 immunostaining counterstained with hematoxylin of a representative 7 dpc ISs from ONA-treated rats. Details are shown in higher magnification. b, border area of resorpted IS; c, center area of resorpted IS. Bar = 500 μm, magnification bar = 100 μm.
(TIF)

S5 Fig Kinetics of decidualization markers, CCND3 and DESMIN, during early pregnancy. Kinetics of Cyclin D3 and DESMIN protein expression during decidualization. Western blots from Non Pregnant (0) and 2, 4, 6, 7, 8 and 9 days post-coitum (dpc) extracts were analyzed. Each stage of pregnancy DESMIN and CCND3 protein levels relative to GAPDH were divided by the NP corresponding level. Data in graphs represent mean fold change ± SEM from at least three rats/day of pregnancy, a minimum of 2 IS/rat was analyzed. One representative blot is shown for each protein. *, P < 0.05; ***, P < 0.001.
(TIF)

S6 Fig Morphology, CD31 and p-ERK1/2 immunolocalization in 10 dpc ISs after Oil and ONA+ICI at 5 and 6 dpc consecutive injections. Pictures show H&E staining (upper panel) and immunohistochemistry of CD31 (mid panel) and p-ERK1/2 (lower panel) of a representative 10 dpc Oil-treated rat and of different 10 dpc ISs from ONA+ICI treated rats quantified in Fig 5B. AM, antimesometrium; Myo, myometrium. Bar = 500 μm; magnification bar = 100 μm.
(TIF)

S7 Fig Morphology effects of ONA+ICI on 7, 10 and 20 dpc rat implantation sites after 5 and 6 dpc consecutive injections. Pictures show H&E staining of a representative 7, 10 and 20 dpc ISs from ONA+ICI treated rats. AM, antimesometrium; M, mesometrium; J, junctional zone; Myo, myometrium; R, resorption. Bar = 500 μm. Arrow indicates resorpted area.
(TIF)

S1 Table Primer sequences used for qRT-PCR. (TIF)
