# Supplementary figures and images for: A Suppressive Antagonism Evidences Progesterone and Estrogen Receptor Pathway Interaction with Concomitant Regulation of Hand2, Bmp2 and ERK during Early Decidualization
Source: PLoS One. 2015 Apr 21;10(4):e0124756. doi: 10.1371/journal.pone.0124756 (PMC4405574; doi:10.1371/journal.pone.0124756)

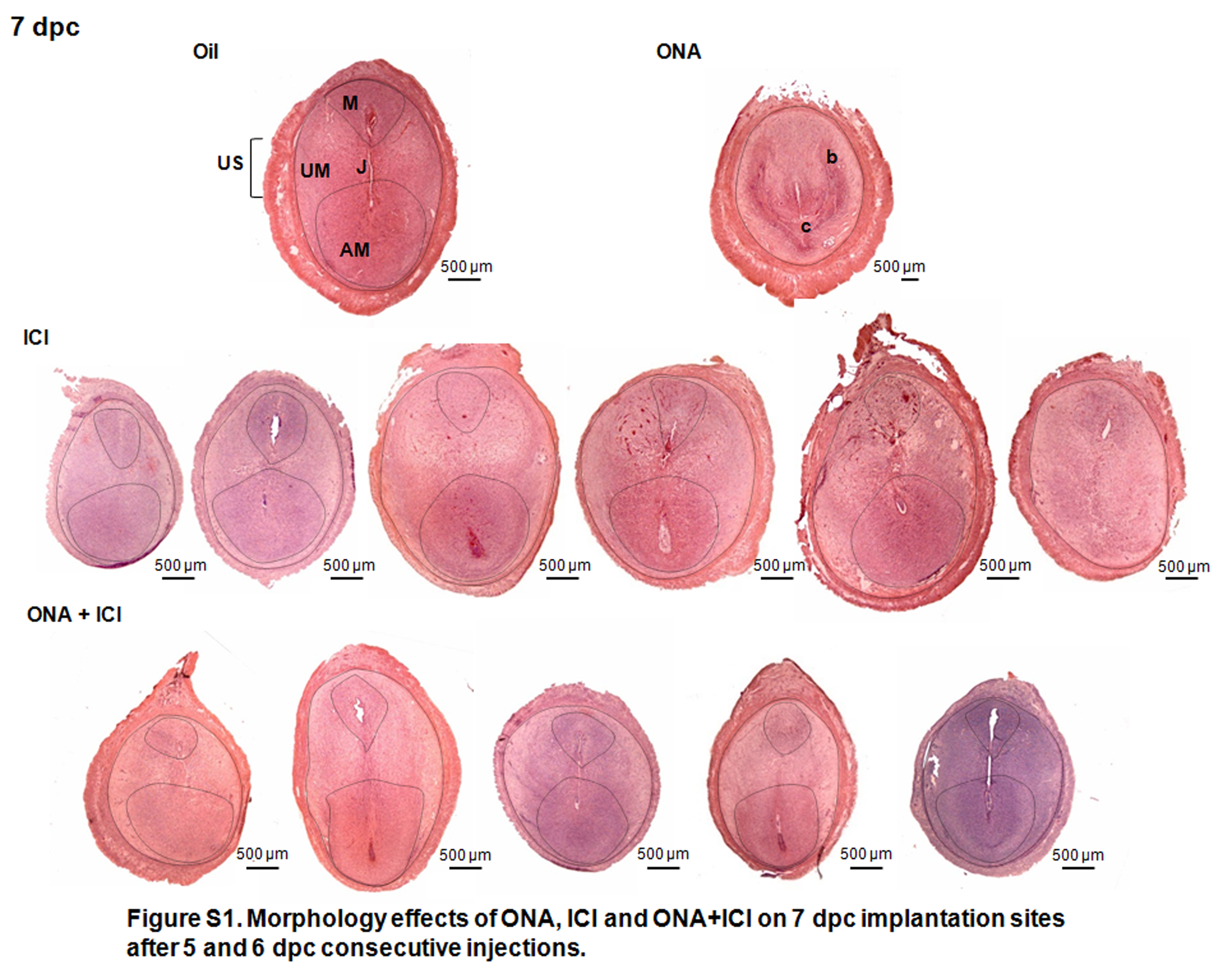

Supplement: S1 Fig — Pictures show H&E staining of 7 dpc IS from Oil, ONA, ICI and ONA+ICI treated rats analyzed and quantified in Fig 1B. Black lines define the decidual areas quantified. AM, antimesometrial decidua; M, mesometrial decidua; J, junctional zone; UM, under myometrium; US, undifferentiated stroma; Myo, myometrium; b, border area of resorpted IS; c, center area of resorpted IS. Bar = 500 μm. (TIF) [file pone.0124756.s001.tif]

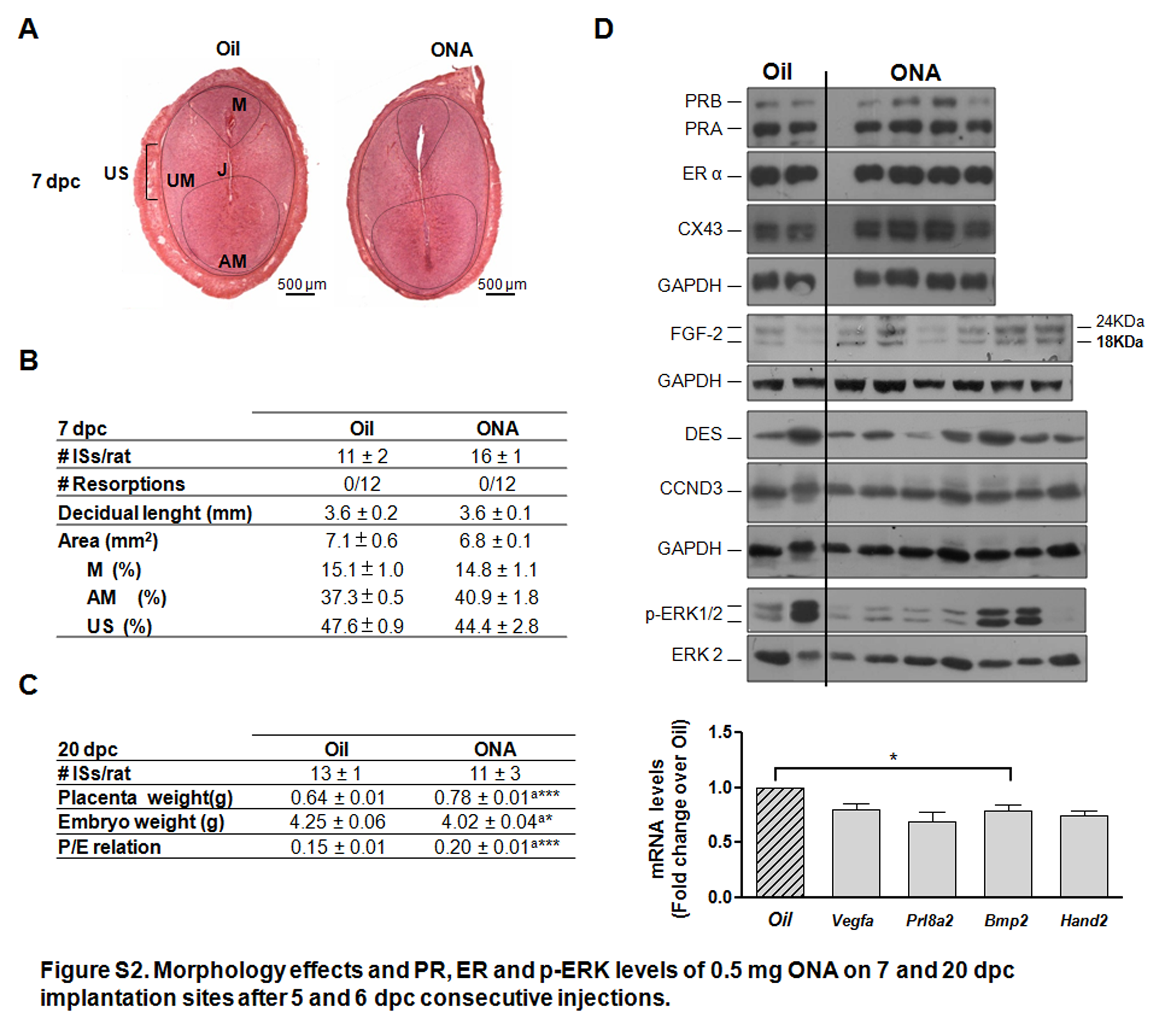

Supplement: S2 Fig — A) Pictures show H&E staining of a representative 7 dpc IS from 0.5 mg ONA treated rats. B) Quantitative analysis of ISs shown in A. Table shows the number of implantation sites/rat, the number of resorptions/total number of ISs analyzed, the mean ± SEM of length and mean ± SEM of total area of decidua and the percentage of tissue areas corresponding to the different regions within the ISs relative to the area of total decidua. Black lines define the decidual areas quantified. At least 3 independent ISs were quantified. C) Quantitative analysis of the effects of 0.5 mg ONA on 20 dpc implantation sites after 5 and 6 dpc consecutive injections. Table shows the number of implantation sites/rat, the mean ± SEM of total area of decidua, the Placenta and Embryo weight and the relation of Placenta weight to Embryo weight. D) Protein expression levels of PRA, PRB, ER α, CX43, FGF-2, DES, CCND3, GAPDH, ERK2 and activated ERK1/2 were analyzed by western blot and mRNA expression levels of Vegfa, Prl8a2, Bmp2 and Hand2 analyzed by qRT-PCR. Data represent mean fold change ± SEM from at least three independent rats/treatment, a minimum of 2 IS/rat was analyzed. *, P < 0.05; ***, P < 0.001; a, statistical differences v. Oil; g, grams. AM, antimesometrium; M, mesometrium; J, junctional zone; UM, under myometrium; US, undifferentiated stroma; Myo, myometrium. Bar = 500 μm. mm, millimeters; mm2, square millimeters; g, grams. (TIF) [file pone.0124756.s002.tif]

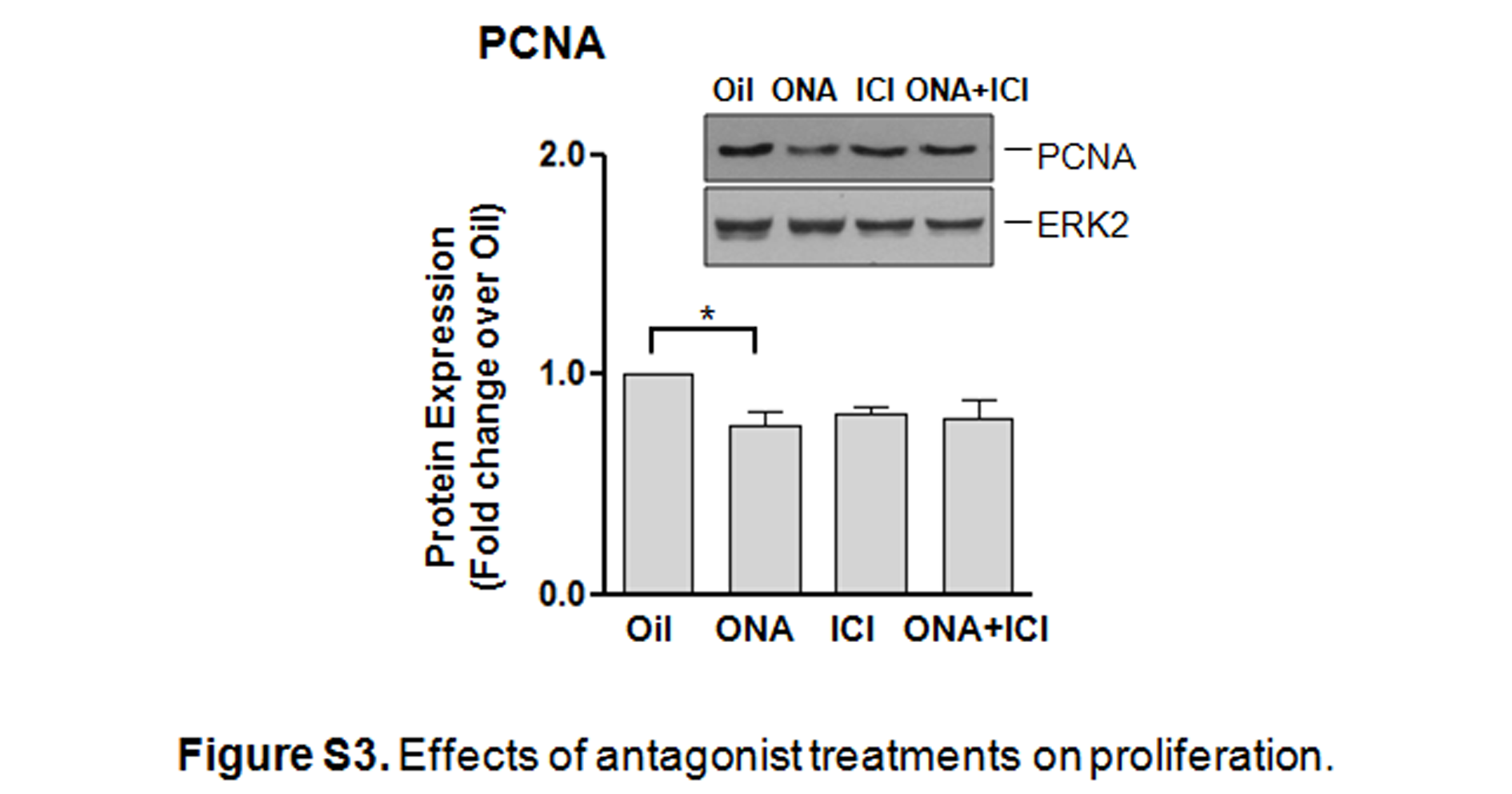

Supplement: S3 Fig — Samples from 7 dpc Oil, ONA, ICI or ONA+ICI treated rats were analyzed for protein expression of PCNA. In each treatment, protein levels of PCNA relative to ERK2 were divided by Oil-treated value. Data represent mean fold change ± SEM from at least three rats/treatment, a minimum of 2 IS/rat was analyzed. Insets show pictures of a representative western blot. *, P < 0.05. (TIF) [file pone.0124756.s003.tif]

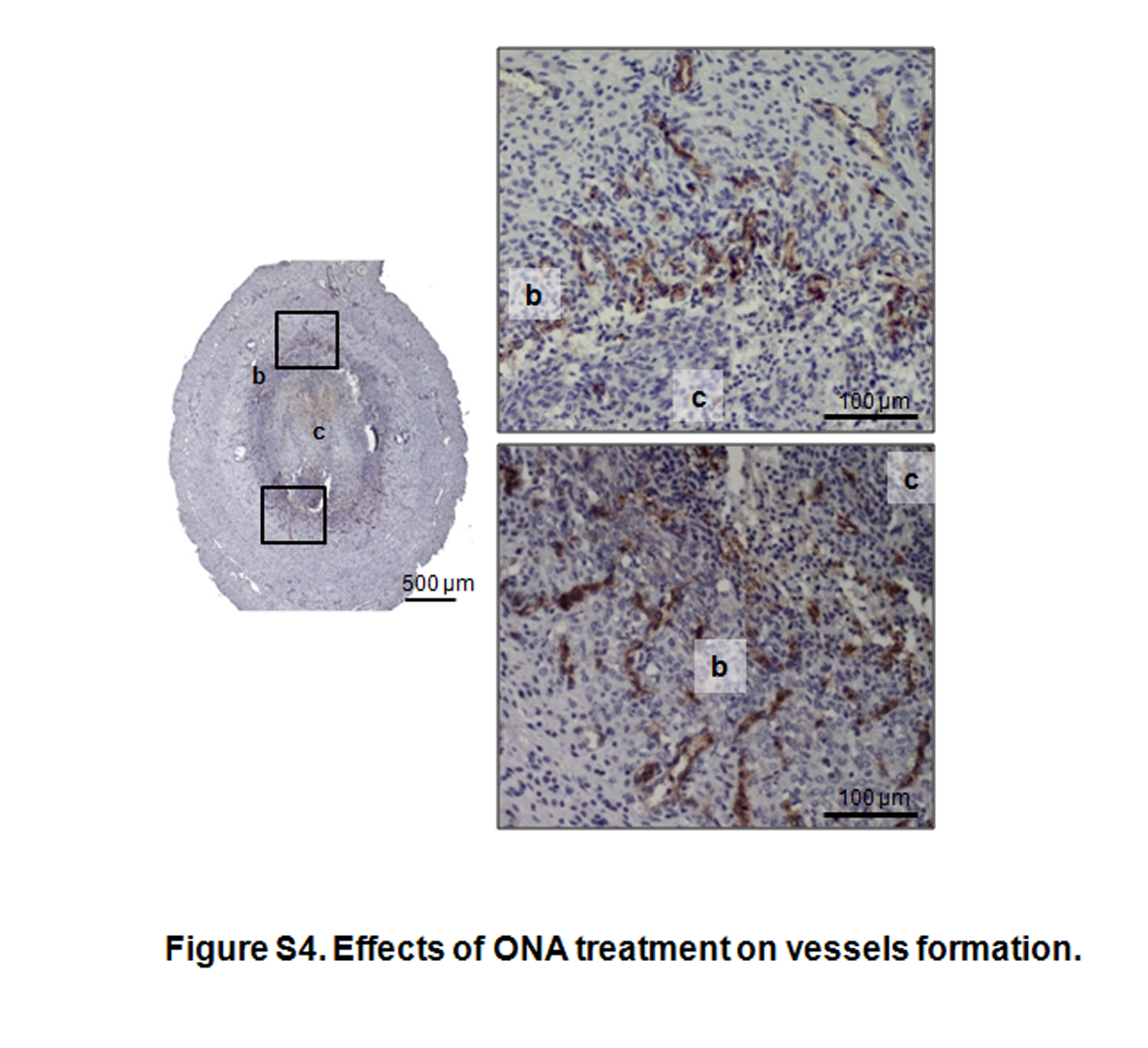

Supplement: S4 Fig — CD31 immunostaining counterstained with hematoxylin of a representative 7 dpc ISs from ONA-treated rats. Details are shown in higher magnification. b, border area of resorpted IS; c, center area of resorpted IS. Bar = 500 μm, magnification bar = 100 μm. (TIF) [file pone.0124756.s004.tif]

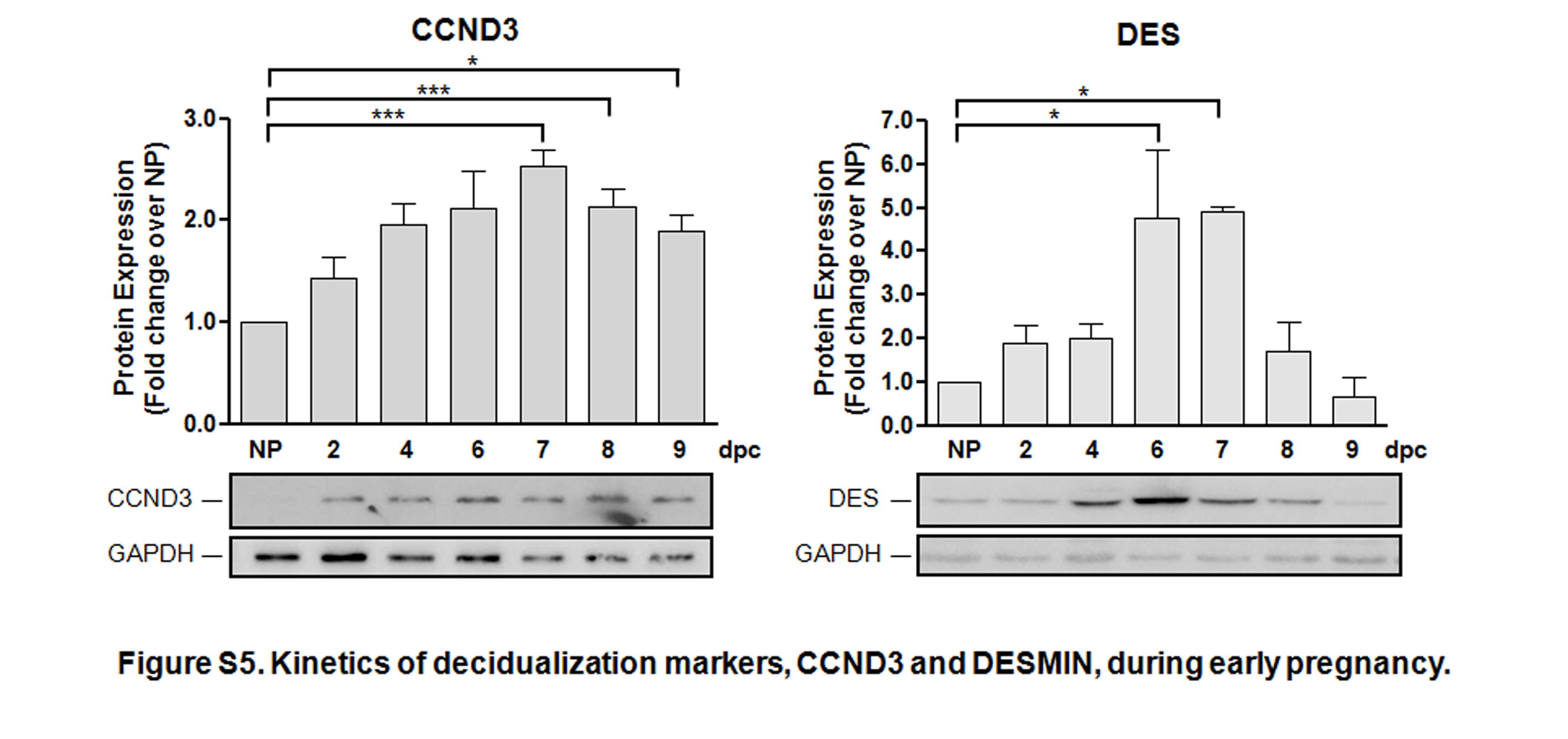

Supplement: S5 Fig — Kinetics of Cyclin D3 and DESMIN protein expression during decidualization. Western blots from Non Pregnant (0) and 2, 4, 6, 7, 8 and 9 days post-coitum (dpc) extracts were analyzed. Each stage of pregnancy DESMIN and CCND3 protein levels relative to GAPDH were divided by the NP corresponding level. Data in graphs represent mean fold change ± SEM from at least three rats/day of pregnancy, a minimum of 2 IS/rat was analyzed. One representative blot is shown for each protein. *, P < 0.05; ***, P < 0.001. (TIF) [file pone.0124756.s005.tif]

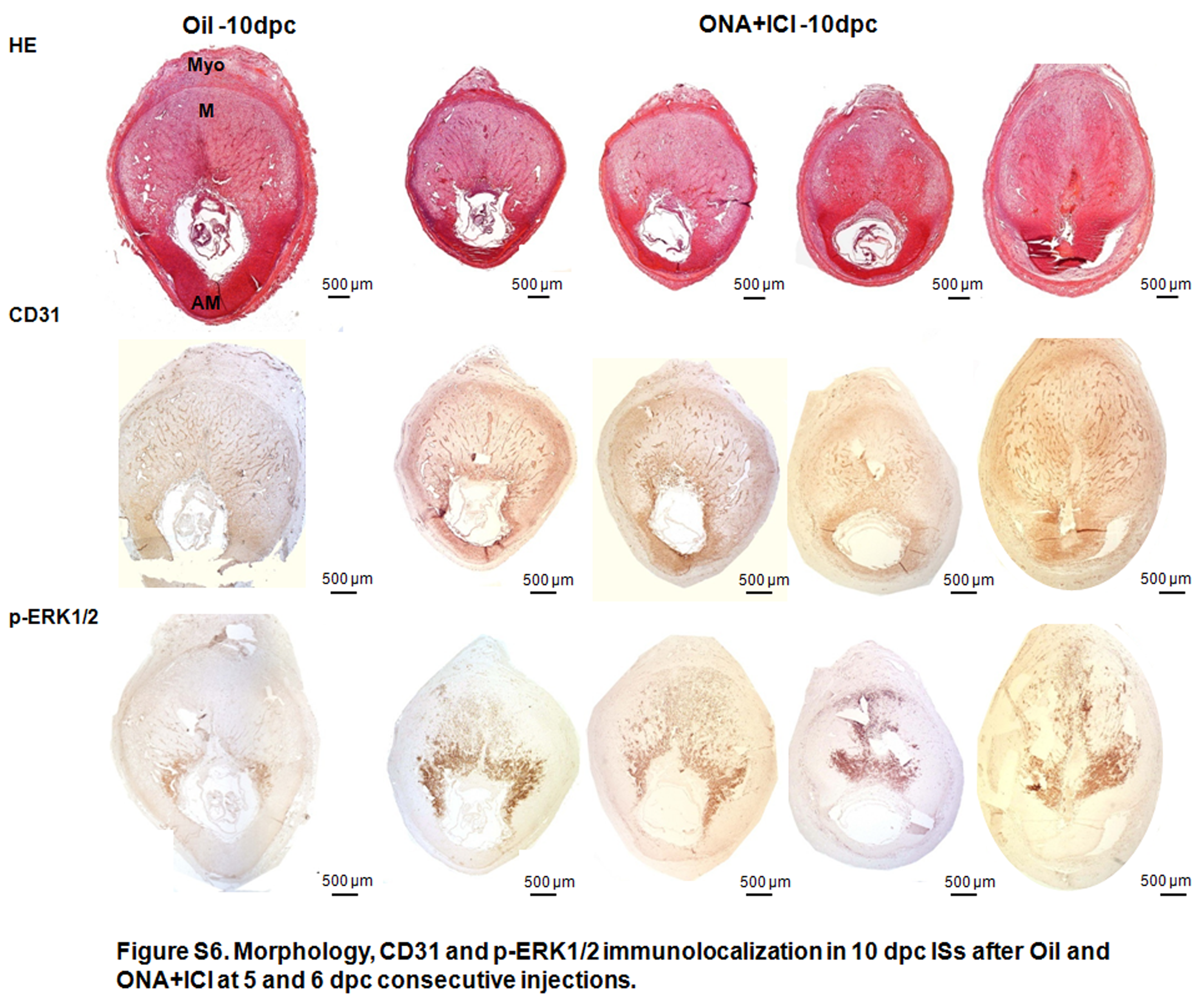

Supplement: S6 Fig — Pictures show H&E staining (upper panel) and immunohistochemistry of CD31 (mid panel) and p-ERK1/2 (lower panel) of a representative 10 dpc Oil-treated rat and of different 10 dpc ISs from ONA+ICI treated rats quantified in Fig 5B. AM, antimesometrium; Myo, myometrium. Bar = 500 μm; magnification bar = 100 μm. (TIF) [file pone.0124756.s006.tif]

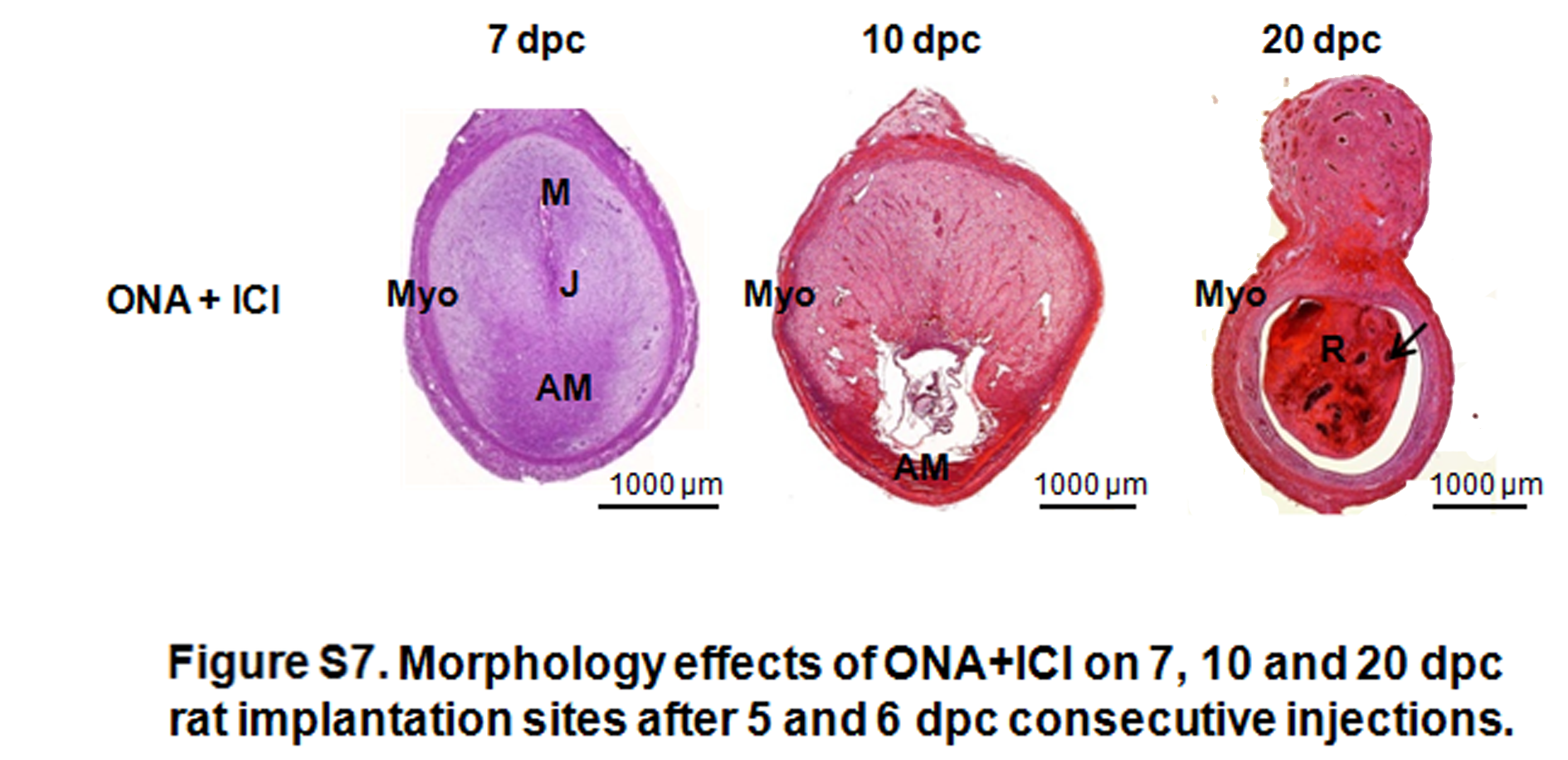

Supplement: S7 Fig — Pictures show H&E staining of a representative 7, 10 and 20 dpc ISs from ONA+ICI treated rats. AM, antimesometrium; M, mesometrium; J, junctional zone; Myo, myometrium; R, resorption. Bar = 500 μm. Arrow indicates resorpted area. (TIF) [file pone.0124756.s007.tif]

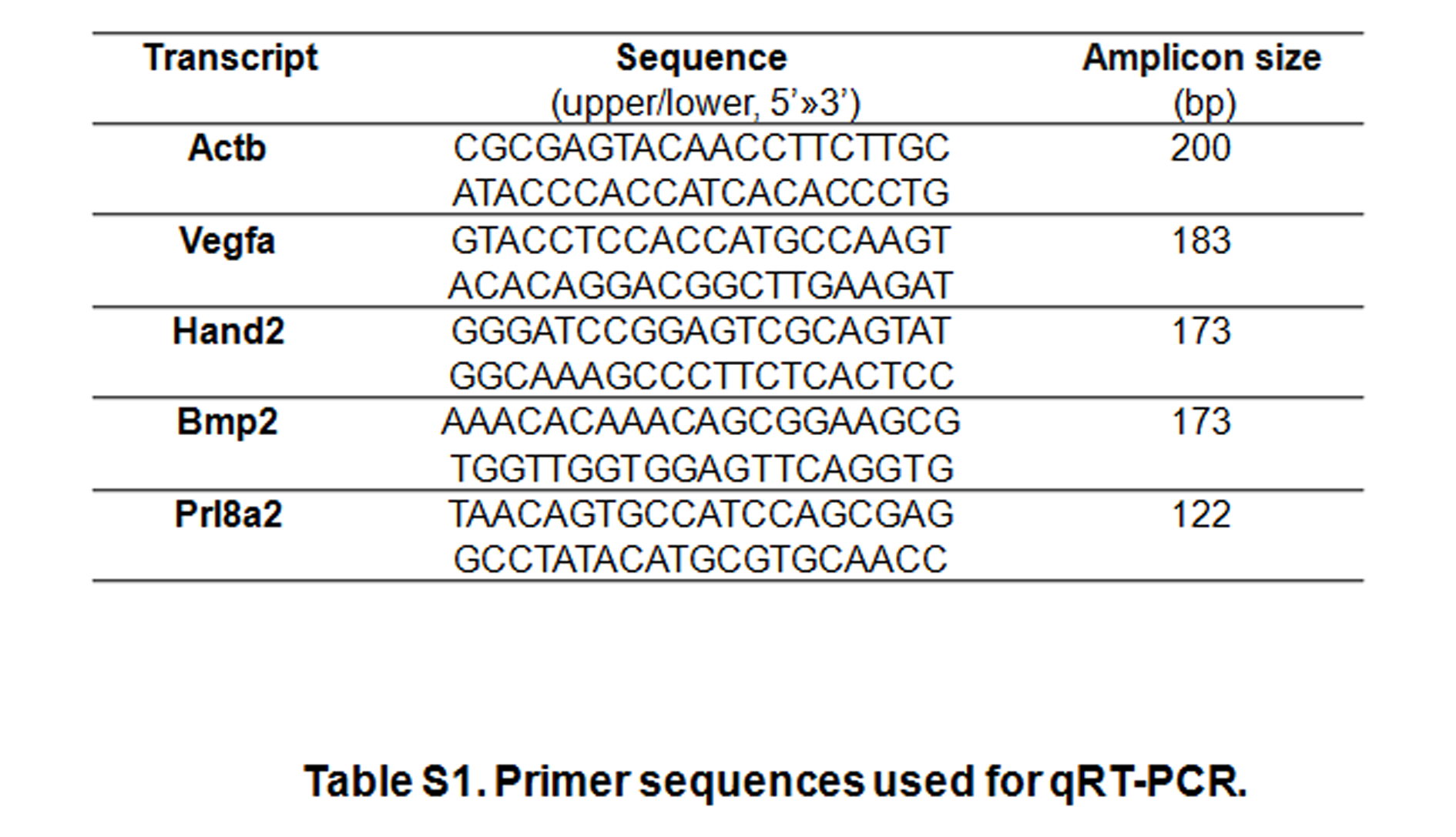

Supplement: S1 Table — (TIF) [file pone.0124756.s008.tif]
